# PPAR Agonists and Cardiovascular Disease in Diabetes

**DOI:** 10.1155/2008/245410

**Published:** 2008-01-02

**Authors:** Anna C. Calkin, Merlin C. Thomas

**Affiliations:** JDRF Center for Diabetes Complications, Baker Heart Research Institute, Melbourne, VIC 3004, Australia

## Abstract

Peroxisome proliferators activated receptors (PPARs) are ligand-activated nuclear transcription factors that play important roles in lipid and glucose homeostasis. To the extent that PPAR agonists improve diabetic dyslipidaemia and insulin resistance, these agents have been considered to reduce cardiovascular risk. However, data from murine models suggests that PPAR agonists also have independent anti-atherosclerotic actions, including the suppression of vascular inflammation, oxidative stress, and activation of the renin angiotensin system. Many of these potentially anti-atherosclerotic effects are thought to be mediated by transrepression of nuclear factor-kB, STAT, and activator protein-1 dependent pathways. In recent clinical trials, PPARα agonists have been shown to be effective in the primary prevention of cardiovascular events, while their cardiovascular benefit in patients with established cardiovascular disease remains equivocal. However, the use of PPARγ agonists, and more recently dual PPARα/γ coagonists, has been associated with an excess in cardiovascular events, possibly reflecting unrecognised fluid retention with potent agonists of the PPARγ receptor. Newer pan agonists, which retain their anti-atherosclerotic activity without weight gain, may provide one solution to this problem. However, the complex biologic effects of the PPARs may mean that only vascular targeted agents or pure transrepressors will realise the goal of preventing atherosclerotic vascular disease.

## 1. INTRODUCTION

Cardiovascular complications are the leading cause of premature mortality in patients with diabetes [1]. While classical risk factors for cardiovascular disease (CVD), such as smoking, cholesterol, and hypertension, operate in persons both with and without diabetes, the absolute risk of death is 2–4 times greater in patients with diabetes [2] and progressively larger with each additional risk factor [3]. Moreover, CVD, cerebrovascular diseases, and peripheral vascular diseases significantly contribute to the morbidity in individuals with diabetes [1]. Ultimately, these macrovascular complications will develop in more than half of the diabetic population [1]. In primary care, over a third of all patients presenting with type 2 diabetes have an overt history of CVD, with a similar number again likely to have undiagnosed macrovascular disease [4]. Consequently, a key component (and some would argue the most important component) in the management of diabetes is the primary and secondary prevention of cardiovascular events.

Diabetes is said to act as an amplifier of cardiovascular risk leading to the increased incidence, size, and complexity of atherosclerotic plaques [5, 6]. A number of components contribute to accelerated atherosclerosis in diabetes. Diabetic dyslipidaemia and insulin resistance significantly contribute to the development and progression of macrovascular disease in diabetes. In addition, inflammation, oxidative stress, enhanced matrix metalloproteinase activity, activation of the local renin angiotensin system (RAS), and the accumulation of advanced glycation end-products (AGEs) in the diabetic vasculature also act to enhance atherogenesis and impair plaque stability. Significantly, each of these pathways may be modified by the activity of peroxisome proliferator-activated receptors (PPARs), ligand-activated nuclear transcription factors with a diverse range of metabolic functions [7–11]. This review will examine the actions of PPARs in diabetes-associated atherosclerosis and explore the recent controversies surrounding the actions of PPAR agonists on CVD in patients with diabetes.

## 2. PEROXISOME PROLIFERATOR-ACTIVATED RECEPTORS (PPARs) 

**Figure 1:**
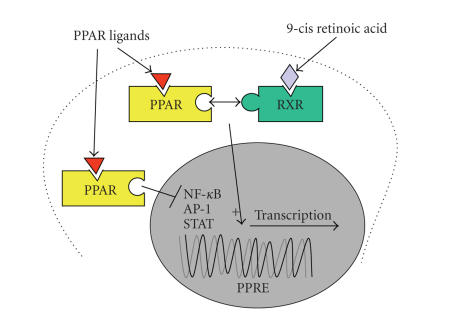
Transactivation and transrepression effects of peroxisome proliferator-activated receptors.

PPARs are nuclear transcription factors that have complex biological effects, resulting from the transactivation or transrepression of dozens of genes that play important roles in glucose and lipid homeostasis [12]. Transactivation effects require ligand-activated dimerisation of PPAR with the retinoid X receptor (RXR), followed by translocation of the PPAR : RXR heterodimer complex to the nucleus, whereupon it binds to PPAR response elements of target genes and induces their expression [12]. *Transrepression* effects are mediated via interference with transcription factors such as activator protein-1 (AP-1) and nuclear factor-*κ*B (NF-*κ*B) [13] (see Figure 1). In addition, conformational remodelling of the PPAR receptor that follows ligand binding results in the release of co-repressor molecules. The relative importance of activation versus repression pathways for the *in vivo* actions of PPAR agonists remains to be established. Moreover, there is evidence that all PPAR ligands do not stimulate transactivation and transrepression pathways to a similar extent, meaning that different agents of the same class may have potentially disparate effects [14, 15].

Three different PPAR isoforms have been identified in humans. These share similar structural organization and sequence homology. However, these isoforms possess distinct functions, and vary in their ligand affinity, expression, and activity in different metabolic pathways.

## 3. PEROXISOME PROLIFERATOR-ACTIVATED RECEPTOR ALPHA (PPAR*α*)

PPAR*α* is highly expressed in the vasculature, including the endothelial cells [16, 17], smooth muscle cells [18], and macrophages [19]. Activation of PPAR*α* leads to modulation of lipid metabolism, including transcription of apolipoprotein A1 (apoA1) [20] and apolipoprotein AII [21], resulting in increased levels of “cardioprotective” high-density lipoprotein (HDL) cholesterol. Uptake of HDL cholesterol is also increased via the upregulation of CLA-1/SR-B1 [22]. *β*-oxidation and lipoprotein lipase (LPL) activity are also stimulated following activation of PPAR*α*. This leads to a decrease in triglycerides and free fatty acids, and levels of apolipoprotein CIII, which inhibits LPL-mediated breakdown of triglycerides, further resulting in lower triglyceride levels [23]. Finally, low-density lipoprotein (LDL) cholesterol particles are shifted from a small, dense to a large, buoyant form to create particles that are less atherogenic and more easily cleared [24]. The natural ligands of PPAR*α* include prostaglandins, leukotrienes, and medium- and long-chain free fatty acids such as eicosapentaenoic acid and docosahexenoic acid [25, 26]. Synthetic ligands of this receptor are utilized in the management of dyslipidaemia [27], and include members of the fibrate drug class (e.g., gemfibrozil, clofibrate, fenofibrate, and bezafibrate).

## 4. PEROXISOME PROLIFERATOR-ACTIVATED RECEPTOR DELTA (PPAR*γ*)

PPAR*γ* is largely expressed in adipose tissue as well as in skeletal muscle, sites where this PPAR isoform exerts much of its metabolic actions [28]. However, PPAR*γ* is also expressed locally in the vasculature, including the endothelial cells [17], smooth muscle cells [29], and macrophages [13, 19]. Broadly, PPAR*γ* activation results in increased sensitivity to the metabolic actions of insulin by reversing lipotoxicity-induced insulin resistance. PPAR*γ* activation has also been shown to rejuvenate pancreatic *β*-cell function resulting in their improved function [30]. In adipose tissue, activation of PPAR*γ* leads to differentiation of adipocytes, making them more able to uptake fatty acids, in turn, sparing other metabolic tissue such as skeletal muscle and liver [28]. In addition, PPAR*γ* agonists increase the expression and activity of glucose transporter-4 and phosphatidyl-3-kinase [31, 32]. The natural ligands of PPAR*γ* include prostaglandins, such as 15-deoxy-(12,14)-prostaglandinJ_2_, and fatty acids including linoleic and arachidonic acids [32]. Synthetic ligands of PPAR*γ* include the thiazolidinedione drug class (e.g., rosiglitazone and pioglitazone). Some other drugs also have partial agonist activity at the PPAR*γ* receptor, including the AT_1_ receptor antagonist, telmisartan [33].

## 5. PEROXISOME PROLIFERATOR-ACTIVATED RECEPTOR DELTA (PPAR*β*/*δ*)

The PPAR delta isoform (also known as beta) is the most widely distributed of the PPARs, with expression seen in most tissues including the vasculature [34]. Unlike the other PPARs, PPAR*β*/*δ*-RXR heterodimers bind to consensus PPAR DNA response elements in the absence of a ligand, and repress target gene expression indirectly by recruiting co-repressors [35]. Following ligand activation, the co-repressor complex is disrupted, leading to enhanced PPAR*β*/*δ* target gene expression by both ligand-induced transcriptional activation and transcriptional derepression. In addition, repressor molecules, such as BCL-6, are liberated on ligand binding, leading to the repression of other pathways, such as inflammation and the transcriptional activity of PPAR*α* and PPAR*γ* [36, 37]. Because of its wide tissue expression, it was initially suggested that PPAR*β*/*δ* might simply serve a house-keeping role. However, more recent data suggest that PPAR*β*/*δ* can play an important role in wound healing, inflammatory responses, and lipid metabolism [34, 36]. For example, PPAR*β*/*δ* activation has been shown to increase HDL cholesterol levels like PPAR*α* ligands, as well as mediate insulin-sensitising and glucose lowering effects like PPAR*γ* [34, 38]. PPAR*β*/*δ* deficient macrophages also show reduced recruitment, which may be particularly important for plaque stability [36] (see below). PPAR*β*/*δ* is activated by a large variety of ligands, such as fatty acids and eiconsanoids including prostaglandin A1, although the major natural ligand remains to be established [25]. Synthetic agonists with nanomolar affinities for PPAR*β*/*δ* have also been generated, although none are currently used in clinical practice. Interestingly, the physiological action of these agonists in experimental atherosclerosis is similar to the phenotype observed in PPAR*β*/*δ* knockout mice [36, 39], consistent with the important actions of transcriptional de-repression following activation of PPAR*β*/*δ*.

## 6. PPAR AGONISTS AND DIABETIC DYSLIPIDAEMIA

Diabetic dyslipidaemia is a major reversible risk factor for the prevention of CVD in individuals with diabetes [40]. A range of quantitative and qualitative lipid and lipoprotein abnormalities are observed in patients with diabetes [41]. The main components of diabetic dyslipidaemia are excessive postprandial lipaemia associated with increased plasma triglyceride [42], due to the accumulation of very low-density lipoprotein (VLDL), chylomicron remnants, and intermediate density lipoprotein particles in the plasma. This is thought to reflect both the overproduction of triglyceride-rich VLDL (due to increased flux of free fatty acids and hepatic resistance to the effects of insulin), together with reduced catabolism (associated with reduced LPL activity) [43]. HDL cholesterol levels are invariably reduced in patients with type 2 diabetes, reflecting increased catabolism of HDL particles [44]. In addition, HDL particles become enriched with triglyceride, in an attempt to cope with an increased VLDL burden. Although LDL cholesterol levels in patients with type 2 diabetes are often within the normal range, there remain significant disturbances in LDL metabolism in diabetes. For example, LDL production is significantly reduced, while impaired turnover of LDL particles promotes glycoxidative modification of lipoprotein particles and cholesterol deposition in the arterial wall [45–47]. Diabetes is also associated with the accumulation of small dense, triglyceride-rich, LDL particles that have an increased atherogenic potential [24].

As noted above, both PPAR agonists are able to significantly modify circulating lipid levels, and therein reduce cardiovascular risk in patients with diabetes [27, 48]. In particular, the use of fibrates in patients with diabetes increases HDL cholesterol, decreases triglyceride levels, and shifts LDL cholesterol distribution toward larger, less atherogenic particles [24, 27, 49]. PPAR*γ* agonists also stimulate reverse cholesterol transport [50, 51] and have beneficial effects on the low HDL cholesterol levels and elevated triglyceride levels that characterize diabetic dyslipidaemia. However, thiazolidinediones can also modestly increase LDL cholesterol levels in some patients [48]. PPAR*β*/*δ* agonists are able to increase HDL cholesterol levels and improve postprandial triglyceride clearance [52].

## 7. PPAR AGONISTS AND INSULIN RESISTANCE

While glycemic control is important for the prevention of microvascular complications, its role in the development of atherosclerotic vascular disease is less clear [53]. For example, in the UKPDS study, macrovascular outcomes were not correlated with HbA_1c_. However, CVD was reduced in patients that received the insulin sensitizer, metformin, when compared to equivalent glycemic control achieved by sulphonylureas or insulin therapy [54]. This led to the hypothesis that insulin sensitivity may itself play an important role in the development of macrovascular disease, and that agents that reduce insulin resistance, such as metformin and PPAR*γ* agonists, by extension, may have particular benefits in the management of type 2 diabetes [55]. Certainly, resistance to the actions of insulin is strongly associated with CVD in patients with diabetes. To the extent that insulin resistance is linked to chronic hyperglycaemia, dyslipidaemia, inflammation, and hypertension as part of the metabolic syndrome, this association is not surprising. However, it is now clear that insulin also has direct actions in the vasculature that influence the development and progression of atherosclerotic disease. For example, in diabetic tissues, selective insulin resistance in the PI-3-kinase signaling pathway leads to reduced synthesis of nitric oxide, impaired metabolic control, and compensatory hyperinsulinaemia. At the same time, insulin signaling, via extracellular signal regulated kinase-(ERK) dependent pathways, is relatively unaffected in diabetes, meaning that hyperinsulinaemia is able to stimulate the expression of endothelin and other pathogenic mediators, tipping the balance of insulin’s actions in favor of abnormal vasoreactivity, angiogenesis, and other pathways implicated in atherosclerosis [56, 57]. In addition, preferential impairment of non-oxidative glucose metabolism in diabetes leads to increased intracellular formation of AGEs and oxidative stress. Nonetheless, while it is conceivable that improvements in insulin sensitivity may have beneficial vascular effects in diabetes, the fact that PPAR agonists retain their anti-atherosclerotic activity in the absence of insulin [9] suggests that other (direct) actions may also be important for their anti-atherosclerotic activities.

## 8. THE POTENTIAL DIRECT ANTI-ATHEROSCLEROTIC ACTIONS OF PPAR AGONISTS

While improvements in metabolic control and the lipid profile have important effects on CVD in patients with diabetes, it is becoming increasingly clear that PPAR agonists have a range of independent actions on the vascular wall which impact on atherogenesis. In particular, pre-clinical studies demonstrate ligand-dependent PPAR activation is able to reduce the development and progression of atherosclerotic lesions in a range of experimental models, without needing to normalise dyslipidaemia and hyperglycaemia, or improve insulin resistance [58]. For example, studies from our group demonstrated that treatment with the PPAR*α* agonist, gemfibrozil, was able to prevent the accumulation of atherosclerotic plaque in apolipoprotein E (apoE) knockout (KO) mice, a model in which PPAR*α* agonists have no effect on severe dyslipidaemia [8]. Similarly, treatment with the PPAR*γ* agonist, rosiglitazone, in insulinopenic diabetic apoE KO mice was also associated with a reduction in aortic atherosclerosis [9], in the absence of insulin sensitization or improvement in glucose levels. Finally, treatment with a PPAR*β*/*δ* agonist, GW0742X, has also been shown to attenuate atherosclerosis in LDL receptor KO mice, in the absence of changes in plasma lipids [39]. Taken together, these studies point to possible direct effects of PPAR agonists on the vasculature that impedes pathogenic pathways implicated in the development of atherosclerosis, including inflammation, oxidative stress, metalloprotease activity, AGE accumulation, and activation of the RAS.

## 9. PPAR AGONISTS AND VASCULAR INFLAMMATION

Inflammation plays a key role in the development and progression of atherosclerotic vascular disease. Inflammatory cells are a major component of early atherosclerotic lesions, and inflammatory cytokines and chemokines accelerate plaque accumulation. Some of the earliest changes involve the activation of endothelial cells, which then express adhesion molecules such as vascular-cell adhesion molecule 1 (VCAM-1) [59], encouraging leucocyte recruitment, the production of chemokines, and further inflammation. Activation of PPAR receptors has also been strongly linked to this early inflammatory response. PPAR***α***,*γ* and *β*/*δ* agonists reduce the expression of adhesion molecules, such as VCAM-1, on the surface of cytokine-activated endothelial cells, as well as reduce macrophage infiltration within atherosclerotic plaque [8, 9, 60, 61]. PPAR agonists also reduce the production of inflammatory cytokines including tumour necrosis factor (TNF)-*α*, IL-6, and IL-1*β* [7, 18]. PPAR*α* activation indirectly modulates inflammatory components in HDL, such as apoA1, serum amyloid A, and paraoxonase-1 [62]. Thiazolidinediones are also able to inhibit endothelial cell activation [63] and indirectly alter systemic inflammation by actions in adipose tissue, reducing the production of pro-atherogenic adipokines including TNF-*α* and resisting [64]. PPAR*β*/*δ* may also have important anti-inflammatory actions. For example, in LDLR KO mice treatment with the PPAR*β*/*δ* agonist, GW0742X, was associated with a marked attenuation of atherosclerosis, with a concomitant decrease in monocyte chemoattractant protein (MCP)-1 and intercellular adhesion molecule (ICAM)-1 [39].

## 10. PPAR AGONISTS AND OXIDATIVE STRESS

Oxidative stress is thought to be a key mediator of atherosclerosis, contributing to the upregulation of adhesion molecules [65], acceleration of foam cell formation, and a reduction in plaque stability [66]. PPAR agonists are also able to modulate oxidative stress in vascular tissues. PPAR*α* activation reduces the expression of the pro-oxidant NAD(P)H subunit p22phox, and increases endothelial expression of the anti-oxidant, CuZn superoxide dismutase [67]. PPAR*γ* agonists also have potent anti-oxidant activity in human endothelial cells [67], hypercholesterolemic rabbits [68], and obese subjects [69]. Studies from our laboratory have shown that treatment of diabetic animals with either a PPAR*α* or a PPAR*γ* agonist is associated with a reduction in vascular superoxide production, together with reduced gene expression of the NAD(P)H oxidase subunits p47phox and gp91phox observed in the aorta of diabetic apoE KO mice [8, 9] (see Figure 3).

## 11. PPAR AGONISTS ANDMATRIX METALLOPROTEINASES

Atherosclerotic plaque rupture, with subsequent occlusive thrombosis, is the underlying cause of sudden cardiac events. Matrix metalloproteinases (MMPs) are thought to mediate the progression of atherosclerotic lesions to an unstable phenotype that is more prone to rupture, through the destruction of the overlying fibrous cap. PPAR agonists may promote plaque stability by reducing the production of MMPs from monocytes/macrophages and vascular smooth muscle cells [70]. Our group has recently demonstrated that gemfibrozil treatment was associated with attenuation of diabetes-associated MMP-2 and MMP-9 gene expression in aorta of diabetic apoE KO mice [8]. Furthermore, studies in patients with type 2 diabetes and CVD have shown that treatment with a PPAR*γ* agonist is associated with a reduction in plasma MMP-9 levels [11].

## 12. PPARs AND ADVANCED GLYCATION END-PRODUCTS

The accumulation of AGEs, as a result of hyperglycaemia, dyslipidaemia, and oxidative stress in diabetes, contributes to the development and progression of vascular disease in diabetes [71, 72]. AGEs accumulate in many diabetic tissues [73], including in atherosclerotic plaques [71]. Their importance as downstream mediators of hyperglycaemia in diabetes has been amply demonstrated by animal studies using inhibitors of advanced glycation to retard the development of atherosclerotic vascular disease without directly influencing plasma glucose levels [71, 74]. Furthermore, dietary excess of AGEs has been shown to accelerate atherosclerosis without affecting glycemic control [75]. Recent studies suggest that, in addition to lowering glucose levels, PPAR*γ* agonists are able to inhibit the formation of AGEs [76]. The mechanism by which PPAR*γ* agonists might reduce AGEs remains to be established, although their anti-oxidant and lipid lowering activities may be relevant to AGE formation and the advanced glycation pathway [10].

## 13. PPARs AND THE RENIN ANGIOTENSIN SYSTEM

The RAS has an important role in the development and progression of diabetic atherosclerosis. For example, our group has demonstrated clear anti-atherosclerotic activity of RAS blockade with an AT_1_ receptor antagonist or an angiotensin converting enzyme (ACE) inhibitor in diabetic apoE KO mice [77, 78]. PPAR activators are known to be negative regulators of the AT_1_ receptor gene. For example, our studies with either rosiglitazone or gemfibrozil resulted in a significant reduction in the vascular expression of the AT_1_ receptor in diabetic apoE KO mice (see Figure 4). At least in this model, this repression of AT_1_ receptor expression by PPAR agonists may function, in terms of atherogenesis, in an equivalent manner to angiotensin receptor blockade.

## 14. PPARs AND THE DIABETIC KIDNEY

Chronic kidney disease is a major risk factor for cardiovascular disease in patients with diabetes. For example, myocardial infarction and stroke are 10 times more common in type 1 diabetic patients with kidney disease than those without renal disease [79]. Below the age of 50 years, the excess of mortality from cardiovascular disease is almost entirely confined to patients with diabetic nephropathy [80]. Equally, in patients with type 2 diabetes, the risk of developing cardiovascular disease is 2-3 times higher in others with microalbuminuria compared to normal albumin excretion. In patients with proteinuria, the risk is increased at least 10-fold [81].

**Figure 2:**
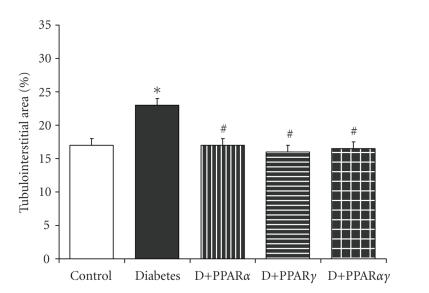
An increase in renal tubulointerstitial area associated with streptozotocin diabetes in apoE KO mice is attenuated following treatment with PPAR*γ* agonist, rosiglitazone, PPAR*α* agonist, gemfibrozil, or the dual PPAR*α*/*γ* agonist, ragaglitazar.

**Figure 3:**
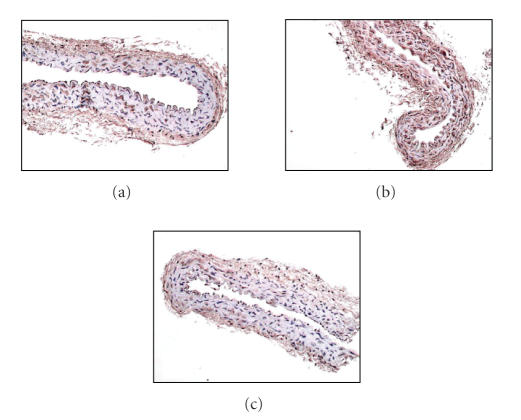
Cross-sections of aorta stained for the NAD(P)H oxidase subunit, p47phox. ApoE KO mouse aorta from (a) control, (b) diabetic, and (c) diabetic + rosiglitazone treated mice.

**Figure 4:**
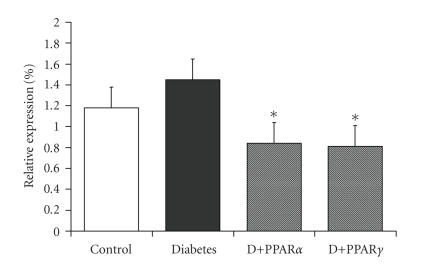
Gene expression of the angiotensin II subtype 1 receptor as assessed by real-time RT-PCR in aorta from apoE knockout mice treated with the PPAR*γ* agonist, rosiglitazone or the PPAR*α* agonist, gemfibrozil for 20 weeks. *P* *<* .05 versus diabetic mice.

PPAR agonists have a number of important actions in the diabetic kidney, which may attenuate renal injury and therein (indirectly) reduce cardiovascular risk. For example, we have shown that albuminuria in streptozotocin-diabetic mice is reduced by treatment with the PPAR*α* agonist, gemfibrozil, the PPAR*γ* agonist, rosiglitazone, or the dual PPAR agonist, compound 3q [82]. In addition, glomerulosclerosis, tubulointerstitial expansion (see Figure 2), and collagen deposition were significantly attenuated. PPAR*γ* agonists may also have beneficial actions on renal hypertrophy in models of experimental diabetes [83–85]. Notably, these renoprotective effects are observed in the absence of changes in glucose or lipid levels, insulin sensitivity, or a reduction in blood pressure, and taken together suggest some independent renoprotective action. Moreover, the finding of similar beneficial effects of PPAR*α* and PPAR*γ* agonists, as well as thiazolidinedione and non-TZD dual agonist compounds, raises the possibility that neither of these agents are working through conventional PPAR*α* and *γ* pathways in this model, but through the transrepression of other transcription factors implicated in diabetic kidney disease including AP-1, signal transducers and activators of transcription 1 (STAT-1) and NF-*κ*B, even in the absence of PPAR receptors [86]. Importantly, these renal benefits have also been observed in clinical trials with PPAR agonists, including the recently completed FIELD trial where a reduction in microalbuminuria was observed in patients treated with fenofibrate [87]. Similarly, in the Diabetes Atherosclerosis Interventional Study, fenofibrate therapy was associated with reduced progression from normal urinary albumin excretion to microalbuminuria in patients with type 2 diabetes [88]. Several previous studies have also demonstrated that thiazolidinediones are able to improve markers of renal structure and function in patients with diabetes [89–91]. However, the cardioprotective benefits of long-term renoprotection observed in these studies remain to be established.

## 15. CLINICAL TRIALS WITH PPAR*α* AGONISTS

A number of clinical studies have shown that treatment with lipid lowering agents is able to prevent adverse CVD outcomes in patients with diabetes. Yet while PPAR*α* agonists are able to reduce lipid levels in patients with diabetes, their clinical efficacy remains controversial, with a number of both positive and equivocal results reported in clinical trials. For example, in the Veterans Affairs High-Density Lipoprotein Intervention Trial (VA-HIT), patients with diabetes treated with gemfibrozil had a reduced risk of a composite end point of coronary heart disease (CHD) death, stroke, or myocardial infarction by 32% and reduced CHD deaths by 41% compared to those with diabetes receiving standard care [92]. Moreover, the clinical benefit derived from fibrates exceeded that attributable to changes in the lipid profile. The Diabetes Atherosclerosis Intervention Study (DAIS) showed that 3 years of treatment with fenofibrate resulted in significant reductions in angiographic progression of atherosclerosis and stenosis (*P* *≤* .03) [27]. Ciprofibrate therapy has also been associated with an increase in flow-mediated dilation in association with an improvement in lipid profile in people with type 2 diabetes [49]. However, in the Helsinki Heart Study, although gemfibrozil reduced the incidence of primary CHD compared with placebo among patients with diabetes (3.4 versus 10.5%), this difference was not statistically significant [93]. Similarly, the recently published Fenofibrate Intervention and Event Lowering in Diabetes (FIELD) study demonstrated a non-significant 11% reduction in the primary end point (coronary artery disease (CAD) death or non-fatal myocardial infarction (MI); *P* = .16) and an 11% reduction in total cardiovascular events (*P* = .035) [87]. However, a 25% reduction in total CVD events and coronary heart disease events was observed in patients without a history of CVD (*P* = .014). This possibly suggests that early and primary therapy with PPAR*α* agonists, comparable to the strategy employed in animal models and shown to be definitively anti-atherosclerotic, may also be beneficial in the clinical setting. In addition, improvements in microvascular outcomes, including a reduction in microalbuminuria in the FIELD study, would be expected to have long-term macrovascular benefits.

## 16. CLINICAL TRIALS WITH PPAR*γ* AGONISTS

Thiazolidinediones have been shown to have a range of positive effects on vascular function in clinical studies. For example, small clinical studies have demonstrated positive effects of thiazolidinediones on cardiovascular parameters such as acetylcholine-mediated dilation [94] and pulse wave velocity [11, 95–97]. Whether such benefits translate to a reduction in cardiovascular risk has been tested in several recent and ongoing clinical trials, although these short-term studies may be inadequate to assess a process like atherosclerosis that takes many decades to evolve.

The Prospective Pioglitazone Clinical Trial in Macrovascular Events (PROACTIVE trial) examined the effect of pioglitazone, taken in addition to conventional therapy for three years, on all-cause mortality, non-fatal MI, stroke, acute coronary syndrome, leg amputation, and coronary or leg revascularisation [98]. While there was a non-significant 10% reduction in this primary outcome *P* = .09, the main secondary endpoint (composed of all-cause mortality, non-fatal MI, and stroke) was reduced by 16% (*P* = .03). However, heart failure and symptomatic oedema due to fluid retention due to PPAR*γ* agonists may have masked any benefit from actions on atherogenesis.

A recent meta-analysis has also been performed to examine the cardiovascular effects of the PPAR*γ* agonist, rosiglitazone, which includes outcome data from 35 trials, such as the large Diabetes Reduction Assessment with Ramipiril and Rosiglitazone Medication (DREAM) trial and the A Diabetes Outcome Prevention Trial (ADOPT) [99–101]. This meta-analysis demonstrated that treatment with rosiglitazone increased the risk for MI by 43% (*P* = .03), and death from cardiovascular causes by 64% (*P* = .06). Whether this finding also reflects increased fluid retention remains to be established.

## 17. THE PROMISE OF DUAL *α*/*γ* PPAR AGONISTS

**Figure 5:**
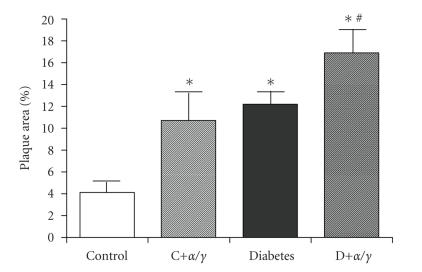
Total aortic plaque area as assessed by an en face approach in apoE knockout mice treated with the dual PPAR*α*/*γ* agonist, *compound 3q* for 20 weeks. *P* *<* .05 versus control mice.

The apparent efficacy of PPAR*α* and PPAR*γ* agonists individually on metabolic control, led to the development of dual PPAR*α*/*γ* agonists, offering the potential of optimising the metabolic and anti-atherosclerotic actions arising from activating both receptors. In general, these agents proved to be more potent agonists of PPAR*γ* than conventional thiazolidinediones and highly effective at improving metabolic parameters. For example, ragaglitazar was more effective at improving glycemic control and attenuating plasma lipid levels than single agonists such as rosiglitazone [102]. Similarly, treatment with muraglitazar in *db/db* mice was more effective at reducing plasma glucose levels than rosiglitazone [103]. Yet despite improved metabolic outcomes, the effects on atherogenesis have been less clear. For example, we found that treatment with the dual PPAR*α*/*γ* agonist, *compound 3q,* was associated with a marked increase in atherosclerosis in control apoE KO mice [104] (see Figure 5), while PPAR*γ* and *α* agonists used alone in this model were protective [8, 9]. This increase in atherosclerotic plaque was observed in control animals despite an improvement in glycemic control and an improvement in lipid profile [104]. Furthermore, plaque accumulation in mice treated with the dual PPAR compound was also associated with a concomitant increase in aortic gene expression of the pro-inflammatory molecules, P-selectin, CD36, VCAM-1, and MCP-1 and increased macrophage infiltration, an effect not seen with the single PPAR agonists, rosiglitazone or gemfibrozil [104]. By contrast, Claudel and colleagues demonstrated that treatment with the dual PPAR*α*/*γ* compound, GW2331, for 11 weeks was more effective at attenuating atherosclerosis in female apoE KO mice than rosiglitazone alone [105]. Similarly, Zuckerman et al. found that LY465608 reduced atherosclerosis in male apoE mice fed a high-fat diet in the absence of changes in plasma total cholesterol levels [106]. More recently, the anti-atherosclerotic actions of tesaglitazar on vascular disease have also been investigated. In apoE* Leiden mice fed a high-fat diet, tesaglitazar was associated with a 92% reduction in aortic atherosclerosis in association with a reduction in macrophages and collagen in lesions [107]. In high-fat fed LDL receptor KO mice treatment with tesaglitazar for 12 weeks was associated also with a decrease in atherosclerosis in female mice in the absence of alterations in cholesterol or triglyceride levels or a reduction in the inflammatory markers serum amyloid A and serum amyloid *P* [108]. The reasons for these conflicting results are unclear. However, it is possible that the different balance of activation of PPAR*α* and *γ* with each of these agents, as well as differential effects on transrepression may have contributed to these disparate findings.

## 18. CLINICAL STUDIES WITH DUAL PPAR AGONISTS

Despite their clear actions as PPAR*γ* and PPAR*α* agonists, and clinical efficacy in terms of lipid and glycemic control [109–116], which were comparable or better than achieved by PPAR agonist alone, recent reports have suggested that dual PPAR*α*/*γ* agonists may also be associated with an increased risk of adverse cardiovascular events when used by individuals with diabetes [116]. In particular, the risk of death, myocardial infarction, stroke, transient ischaemia attack, or CHF was increased by over two-fold (RR 2.62; 95% CI 1.36 to 5.05) in patients with type 2 diabetes receiving the dual agonist, muraglitazar, compared to those receiving a PPAR*γ* agonist (pioglitazone) alone, despite comparable effects on glycemic control [116]. Whether this increase in events is due to an augmentation of atherogenesis, as observed in our pre-clinical models, or the by-product of augmented fluid retention in patients with a stiff vasculature, remains to be established. Certainly, the more potent activation of the PPAR*γ* receptor achieved by dual agonists may lead to clinically important fluid retention in some patients, particularly at high doses or in patients with established congestive heart failure. Nonetheless, even when patients with NYHA III/IV heart failure were excluded from these trials, the muraglitazar treated group still had 13 adjudicated cases of heart failure compared with only one patient in the control group. More recently, the development tesaglitazar has been discontinued due to concerns about increased serum creatinine and decreased glomerular filtration rate [117]. Taken together with reports of toxicity and carcinogenic effects with some of the dual PPAR agonists in pre-clinical studies [118–120], these finding have meant that ongoing evaluation of this class of drug has been delayed and largely superseded by the pan-PPAR agonists (detailed below).

## 19. THE DEVELOPMENT OF PAN-PPAR AGONISTS

The clinical efficacy of PPAR agonists individually have led to the development of chemical ligands with activity across all three receptor isoforms. The potential advantage of such a combination rests in the finding that these so-called “pan-PPAR” agonists retain their broad metabolic activity, without the weight gain associated with PPAR*γ* agonists [121, 122]. Cell culture and pre-clinical studies have also demonstrated the efficacy of pan PPAR agonists in modulating various pathways linked to the development of atherosclerosis [67, 123, 124].

## 20. CLINICAL STUDIES WITH PAN PPAR AGONISTS

There are a small number of pan-PPAR agonists now in the early stages of clinical trials including GW766954, GW625019, PLX-204, and netoglitazone (MCC-555) [125]. These agents have been shown to improve glycaemic and lipid control in a range of settings. While such benefits should confer some cardiovascular benefit, the actions of agents of this class on the development and progression of atherosclerosis in diabetes remain to be established. However, some insight into the possible efficacy of pan PPAR agonists may be inferred from clinical studies using bezafibrate. Although originally classed as a fibrate, bezafibrate is now considered a pan-PPAR agonist, albeit of low potency. Nonetheless, like other PPAR agonists, treatment with bezafibrate significantly raises HDL cholesterol levels, reduces triglycerides, and improves insulin sensitivity in patients with diabetes [126]. In the Bezafibrate Coronary Atherosclerosis Intervention Trial (BECAIT) of dyslipidemic males under the age of 45 who have experienced a previous MI, bezafibrate improved dyslipidaemia, reduced the cumulative coronary event rate (*P* = .02) and slowed the progression of focal coronary atherosclerosis. The St Mary’s, Ealing, Northwick Park Diabetes Cardiovascular Disease Prevention (SENDCAP) trial also demonstrated a reduction in the combined incidence of ischemic change on resting ECG and documented MI [127]. Despite this, they were unable to see any effect of bezafibrate on the progression of coronary of femoral atherosclerosis over the 3 years of the study. By contrast, the Bezafibrate Infarct Prevention (BIP) study demonstrated no significant effect of bezafibrate on fatal or non-fatal MI in those with diabetes [128]. Whether newer and more potent pan-PPAR ligands with differential activation of the various PPAR isoforms will prove to be more beneficial with respect to cardiovascular outcomes remains to be established. In addition, given the actions of PPAR in the transcriptional regulation of an enormous range of genes and pathways, the potential adverse impact of such pan-PPAR activity needs to be carefully studied.

## 21. CONCLUDING REMARKS

Agonists of the PPAR family have represented the most important development in the management of diabetes over the last decade. Despite the promise of improved insulin sensitivity and better lipid control, these agents have not achieved the cardiovascular benefits expected of them. There is little doubt that in experimental models, PPAR agonists have clear and independent anti-atherosclerotic actions, including the suppression of vascular inflammation, oxidative stress, and activation of the renin angiotensin system. Why this has not translated into clinical benefit remains to be fully established. It may be that longer-term follow up of clinical studies will reveal statistically significant results, as the long-term benefits of improved metabolic control are realized. Equally, the complex biological effects of the PPARs in a range of organs may mean that any benefits are offset by unwanted actions that impact on CVD, such as fluid retention, malignancy, renal impairment, or increases in LDL cholesterol. Whether more organ-targeted agonists or pan-PPAR agonists will prove more effective remains to be seen.

 However, the fact that many of the potentially useful vascular effects are thought to be mediated by transrepression of pro-atherogenic signalling pathways, should lead in the future to the development of more selective transrepressors for the prevention and management of cardiovascular disease in diabetes.
